# Elevated expression of MKRN3 in squamous cell carcinoma of the head and neck and its clinical significance

**DOI:** 10.1186/s12935-021-02271-6

**Published:** 2021-10-24

**Authors:** Shuiting Zhang, Chao Liu, Guo Li, Yong Liu, Xingwei Wang, Yuanzheng Qiu

**Affiliations:** 1 Department of Otolaryngology Head and Neck Surgery, Xiangya Hospital, Central South University, Changsha, Hunan People’s Republic of China; 2 Department of Anesthesiology, The Second Xiangya Hospital, Central South University, Changsha, Hunan People’s Republic of China; 3 Otolaryngology Major Disease Research Key Laboratory of Hunan Province, Changsha, Hunan People’s Republic of China; 4 Clinical Research Center for Pharyngolaryngeal Diseases and Voice Disorders in Hunan Province, Changsha, Hunan People’s Republic of China

**Keywords:** Squamous cell carcinoma of the head and neck, MKRN3, P53, Tumorigenesis, Prognostic factor

## Abstract

**Background:**

Squamous cell carcinoma of the head and neck (SCCHN) is one of the most common types of cancer that cause a substantial number of cancer-related deaths. Our previous study has revealed that makorin ring finger protein 3 (MKRN3) may act as a key regulator of the SCCHN tumorigenesis; however, its specific role in SCCHN progression has not been reported.

**Methods:**

The Cancer Genome Atlas (TCGA) data analysis and quantitative polymerase chain reaction (qPCR) were used to quantify the MKRN3 mRNA expression levels in SCCHN; immunohistochemical staining or immunoblotting analyses were performed to detect MKRN3 protein expression. Kaplan–Meier plotter was used to assess the prognostic values of MKRN3 in terms of overall survival and disease-free survival. The expression differences based on various clinicopathological features were evaluated using subgroup analysis and forest map analysis. The regulatory mechanism of MKRN3 was further investigated using gene ontology and Kyoto Encyclopedia of Genes and Genomes analyses. Subsequently, STRING was used to perform a co-expression and enrichment analysis for MKRN3. Homologous modeling, molecular docking, and western blot analyses were performed to investigate the relationship between MKRN3 and its potential target gene P53.

**Results:**

MKRN3 was ectopically expressed between cancerous and noncancerous SCCHN tissues, and its expression level was tightly associated with high T classifications as well as advanced clinical stages. qPCR analysis revealed that MKRN3 was upregulated in the SCCHN cell line. Moreover, Kaplan–Meier and Cox regression analyses indicated that SCCHN patients with high MKRN3 expression had poorer prognosis and that MKRN3 was a potential prognostic marker for SCCHN. Using gene ontology and Kyoto Encyclopedia of Genes and Genomes analyses, we determined that MKRN3 may be involved in the regulation of synthesis and metabolism and cell growth, death and motility, as well as cancer pathways associated with SCCHN progression. Mechanism investigation further revealed that P53, a potential target of MKRN3, may be involved in the SCCHN tumorigenesis mediated by MKRN3.

**Conclusions:**

We performed a comprehensive evaluation of the clinical significance of MKRN3 and explored its underlying mechanisms. We concluded that MKRN3 represents a valuable predictive biomarker and potential therapeutic target in SCCHN.

**Supplementary Information:**

The online version contains supplementary material available at 10.1186/s12935-021-02271-6.

## Background

Squamous cell carcinoma of the head and neck (SCCHN) is a histopathological diagnosis that encompasses epithelial malignancies arising from the paranasal sinuses, nasal cavity, oral cavity, pharynx, and larynx [1]. Despite the advances in oncology treatment strategies, limited improvement in the 5-year survival rate of SCCHN patients has been made in the recent decades [2]. The unfavorable outcome has been primarily attributed to late diagnosis, loco-regional recurrences, and cervical lymph node metastasis [3]. Thus, it is important to investigate the pathogenesis of SCCHN and identify molecular biomarkers with prognostic significance in order to enable optimized therapeutic strategies and prolong patient survival when feasible.

In our previous study, we identified MKRN3 as a candidate regulator of SCCHN tumorigenesis using prediction algorithms [4]. MKRN3, an imprinted gene located on the long arm of chromosome 15 (Prader–Willi region), encodes makorin ring finger protein 3, which is involved in the processes of gene transcription and ubiquitination [5, 6]. The MKRN3 protein has four zinc finger domains including three C3H1 motifs and one C3H4 ring finger with presumed E3 ubiquitin ligase activity [7]. It has been initially shown that MKRN3 deficiency causes central precocious puberty in humans [8]. Thus, the biological functions of MKRN3 were further investigated. Currently, MKRN3 is considered a novel imprinted gene involved in the progression of osteosarcoma [9] and non–small cell lung cancer [10]. However, the specific role of MKRN3 in SCCHN is yet to be determined, particularly with respect to its association with clinical outcomes.

Therefore, our study aimed to investigate the clinical relevance of MKRN3 expression in SCCHN. In the present work, the expression of MKRN3 in SCCHN tissue samples and cell lines has been investigated for the first time. Further experiments were performed to assess whether MKRN3 expression is correlated with clinicopathological parameters and to gain insight into the biological pathways and mechanisms regulated by MKRN3 that are involved in SCCHN pathogenesis.

## Materials and methods

### Data acquisition and processing

**Fig. 1:**
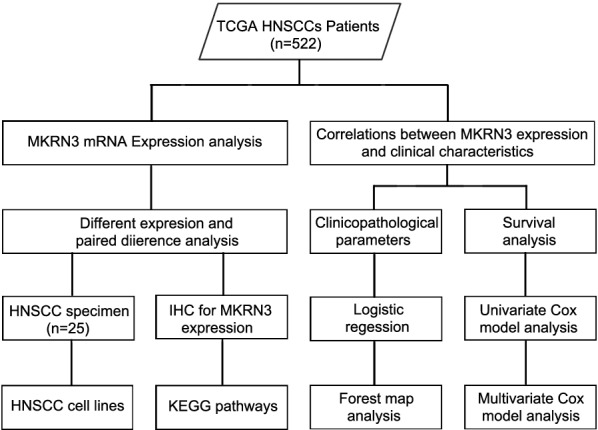
Study flowchart

A workflow chart of this study is shown in Fig. 1. The expression profiles and clinical information of the 522 SCCHN and 44 adjacent noncancerous epithelial samples were obtained from The Cancer Genome Atlas (TCGA) database (Table 1). The MKRN3 expression levels and overall survival are shown in Additional file 6: Table S6; the main clinical and pathological parameters of SCCHN patients are summarized in Table 1. A P-value < 0.05 was set as the significance threshold for differential expression and clinical outcome. The immunohistochemistry (IHC) data was downloaded from Protein Atlas (https://www.proteinatlas.org). IHC evaluation was based on the staining intensity (0–3) and degree (0–4). The MKRN3 protein expression was classified into two groups: high set (score: 4–7) and low set (score: 0–3). Five fields per IHC slides were randomly selected for evaluation and all IHC were independently and blindly assessed as well as scored by investigators.

**Table 1:** Clinical characteristics of TCGA SCCHNs patients

Parameters	No. of patients	Percentage
Age		
< 59	234	44.8
≥ 59	287	55.0
NA	1	0.2
Gender		
Female	137	26.2
Male	385	738
Alcohol		
Yes	348	66.7
No	163	31.2
NA	11	2.1
Smoking		
Yes	297	56.9
No	213	40.8
NA	12	2.3
Histological grade		
G1 + G2	367	70.3
G3 + G4	132	25.3
Gx	18	3.4
NA	5	1.0
Stage		
I + II	118	22.6
III + IV	390	74.7
NA	14	2.7
T classification		
T1 + T2	186	35.6
T3 + T4	274	52.5
Tx	39	7.5
NA	23	4.4
Lymph node metastasis		
N0	176	33.7
N+	246	47.1
N_X_	75	14.4
NA	25	4.8
M classification		
M0	188	36.0
M1	1	0.2
MX	62	11.9
NA	271	51.9

^*^P < 0.05 was considered to be statistical significance

### Cell culture

SCCHN cell lines were kindly provided by Dr. Joseph Califano (University of California, San Diego, USA) and Dr. Zhuo G. Chen (Emory University Winship Cancer Institute,USA) or purchased from ATCC as previously described [11–13]. Human-derived dysplastic oral keratinocytes (DOK), served as a normal cell line, were grown in RPMI 1640 medium (Hyclone, Logan, UT). Tu686 cells were cultured in Dulbecco’s modified Eagle medium (DMEM)/F12 medium (Hyclone). Fadu and CAL27 cells were maintained in DMEM basic medium, whereas JHU011 and Tca8113 cells were kept in RPMI 1640 medium. The culture media were supplemented with 10% fetal bovine serum (FBS) (Gibco, NYC, New York, NY), and all cells were cultured in a humidified incubator at 37 °C and 5% CO_2_.

### SCCHN patient samples

In this study, a total of 25 SCCHN tissues and 23 adjacent normal tissues were collected from patients in Xiangya Hospital, China from September 2015 to December 2017. All samples were collected for quantitative real-time PCR. Additionally, clinicopathologic information of patients that was collected included age at diagnosis, T status, lymph node metastasis, clinical stage, and histology grade. Ages of the 25 SCCHN patients ranged between 38 and 69 years (mean, 56.7 years), and more detailed information are shown in Additional file 5: Table S5. Exclusion criteria for this study were as follows: age < 18 years or age > 75 years; histologically unconfirmed SCCHN; and incomplete clinicopathological parameters. We also excluded patients who had comorbidities and underwent other radiotherapy and chemotherapy strategy.

The study was approved by the Research Ethics Committee of Xiangya Hospital, Central South University, Changsha, China, and all samples were used for analysis under written informed consent from the patients.

### Quantitative reverse transcriptase polymerase chain reaction (qRT-PCR)

The total RNA was isolated from SCCHN tissues and cell lines using the TRIzol reagents (Invitrogen, Carlsbad, CA, USA), and cDNA was reverse transcribed using the All-in-One™ mRNA cDNA synthesis Kit (GeneCopoeia, Rockville, MD, USA) following the manufacturers’ protocols. Relative MKRN3 expression values were calculated using the 2^−△△CT^ method and normalized using the GAPDH expression levels [14, 15]. The primers used are listed in Additional file 1: Table S1.

### Western blotting assay

Total cell protein was lysed and extracted and separated using 8–12% SDS-PAGE gels and transferred onto PVDF membranes (Millipore, Bedford, MA, USA). Next, membranes were blocked with 5% skimmed milk and incubated with primary antibodies at 4 °C overnight. GAPDH was used as the loading control. The protein expression was imaged using Image Lab 4.1 (Bio-Rad, Hercules, CA, USA) with enhanced chemiluminescence reagents. The relevant antibody information are listed in Additional file 2: Table S2.

### Functional enrichment analyses

Protein–protein interaction analysis (PPI), gene ontology (GO) biological process, and Kyoto Encyclopedia of Genes and Genomes (KEGG) pathway enrichment analyses were employed in STRING (http://string-db.org) online database to identify over-represented GO terms in biological processes as well as over-represented KEGG pathway terms. The SWISS-MODEL (https://swissmodel.expasy.org) [16], ZDOCK server (https://zdock.umassmed.edu) [17] and PDBePISA Browser (https://www.ebi.ac.uk) [18] were used for homologous modeling, molecular docking of protein and calculate the Minimum Free Energy. For this analysis, a false discovery rate (FDR) < 0.05 and − log FDR > 1.301 were considered to indicate statistical significance.

### Statistical analysis

All data were analyzed using SPSS version 22.0 (IBM Corp., Armonk, NY, USA). Results are presented as the mean ± standard deviation. Statistical differences between two groups were determined using Student’s *t*-test (for equal variances) or Mann–Whitney *U* test (for unequal variances). Additionally, survival curves were plotted using the Kaplan–Meier method and compared using the log-rank test. P-values < 0.05 were considered statistically significant.

## Results

### Elevated expression of MKRN3 in SCCHN

**Fig. 2:**
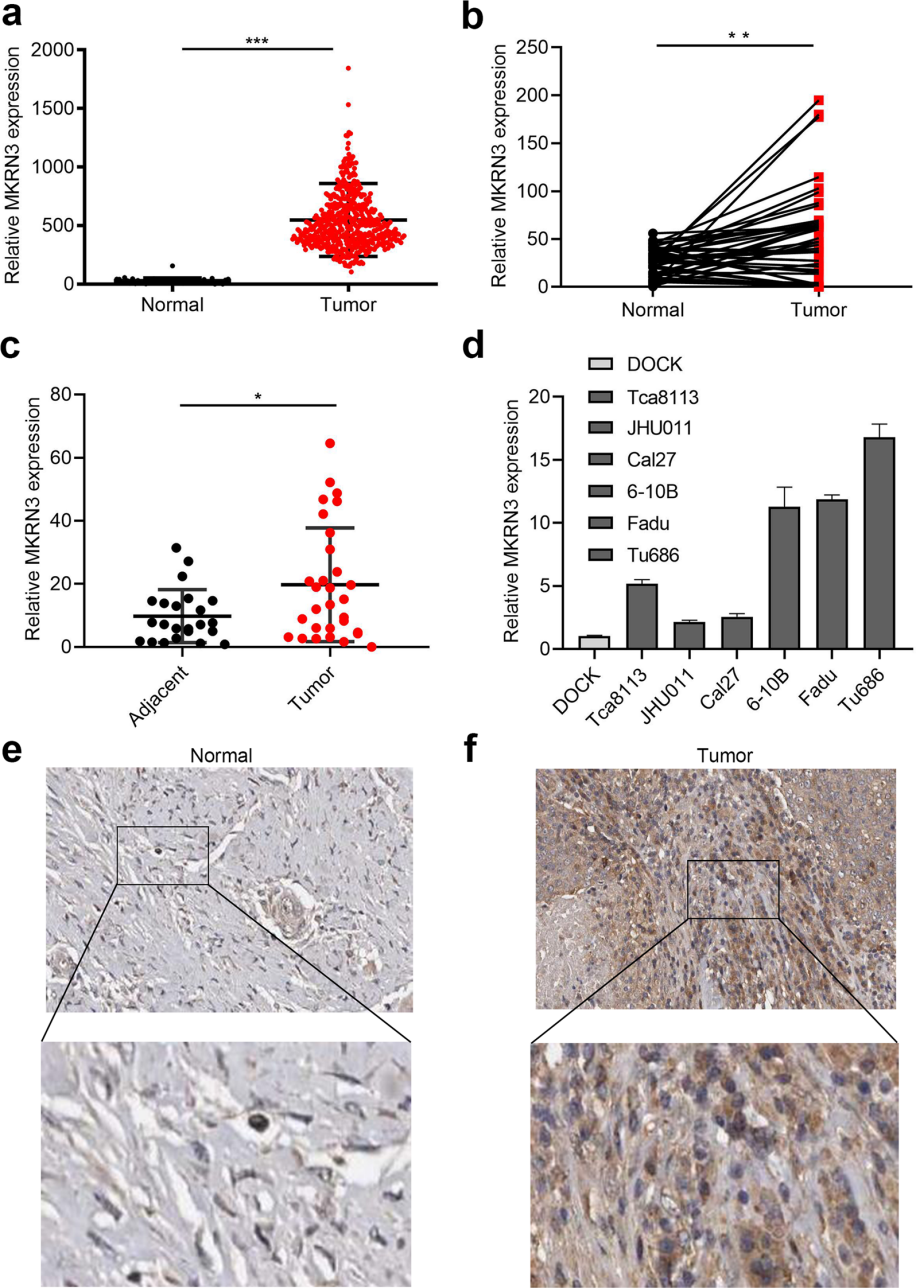
Elevated expression of MKRN3 in SCCHN. **a**, **b** Differential expression of MKRN3 in SCCHN tissue samples according to TCGA data (**a**) and paired difference analyses (**b**). **c** MKRN3 mRNA expression levels in patients with normal tumor tissues. **d** Relative expression level of MKRN3 mRNA in six different SCCHN cell lines and an immortalized non-malignant cell line derived from oral mucosa (DOK). The gene expression was normalized to GAPDH. **e**, **f** Representative immunohistochemical staining shows the expression of the MKRN3 protein in normal (**e**) and SCCHN tissues (**f**) according to TCGA (200× , 400×). Data are presented as the mean ± standard deviation. P-values were calculated using the Student’s *t*-test. *P < 0.05; **P < 0.01; ***P < 0.001. TCGA, The Cancer Genome Atlas

TCGA sequencing data were used to delineate differential expression scatterplots and paired difference analyses. As a result, MKRN3 level was markedly higher in SCCHN samples than in corresponding adjacent noncancerous tissues (Fig. 2a; P < 0.01). This was supported by 40 paired cases (Fig. 2b; P < 0.01). Next, the relative expression of MKRN3 was quantified in the 25 SCCHN samples and in 23 samples of adjacent epithelium using quantitative polymerase chain reaction. Our data revealed that the MKRN3 expression was upregulated in most SCCHN cases compared to those observed in the adjacent tissues (Fig. 2c; P < 0.01). Further analysis also showed that the MKRN3 expression level was greater in SCCHN than in DOK cells (Fig. 2d). Finally, we collected the IHC data for MKRN3 expression in SCCHN specimens to investigate whether the MKRN3 protein level was altered. As shown in Fig. 2e, f, the density and intensity of MKRN3 expression in SCCHN tumor tissues were significantly increased compared to those of para-carcinoma tissues. Together, these results suggest that MKRN3 expression is elevated in SCCHN.

### Correlation between clinicopathological features and MKRN3 expression in SCCHN

As summarized in Table 2, a high expression of MKRN3 was closely associated with smoking, advanced clinical stages, and high T classifications in patients with SCCHN (Table 2; all P < 0.05). These data establish the oncogenic role of MKRN3 in SCCHN.

**Table 2:** Correlations between the expression of *MKRN3* and clinicopathological parameters

Parameters	MKRN3 expression	*t* value		*P*-value^*^
Age				
< 59	66.339 ± 54.931	− 0.318		0.751
≥ 59	67.915 ± 57.543			
Gender				
Female	65.794 ± 60.985	− 0.342	0.733	
Male	67.712 ± 54.665			
Alcohol consumption				
Yes	65.821 ± 53.437	0.044		0.965
No	66.055 ± 59.506			
Smoking				
Yes	66.190 ± 58.305	− 2.311		***0.021***
No	77.953 ± 53.935			
Histological grade				
G1 + G2	70.159 ± 58.499	0.963		0.336
G3 + G4	64.520 ± 47.820			
Stage				
I + II	52.571 ± 39.396	− 2.928		***0.001***
III + IV	71.743 ± 62.453			
T classification				
T1 + T2	62.429 ± 52.840	− 2.348		***0.025***
T3 + T4	75.098 ± 60.669			
Lymph node metastasis				
N0	67.450 ± 54.218	− 0.989		0.560
N +	72.227 ± 61.835			

The bolditalic values represent *P* < 0.05

^*^P < 0.05 was considered to be statistical significance

### A high expression level of MKRN3 predicts a worse prognosis in SCCHN patients

**Fig. 3:**
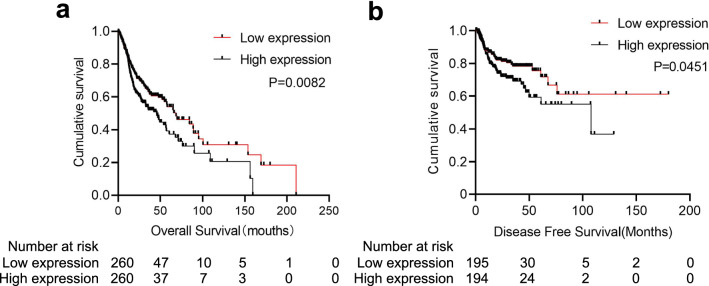
High MKRN3 expression levels predicts a worse prognosis in patients with SCCHN. Kaplan–Meier survival analysis of MKRN3 expression in terms of overall survival (**a**) and disease-free survival (**b**) in SCCHN patients

**Fig. 4:**
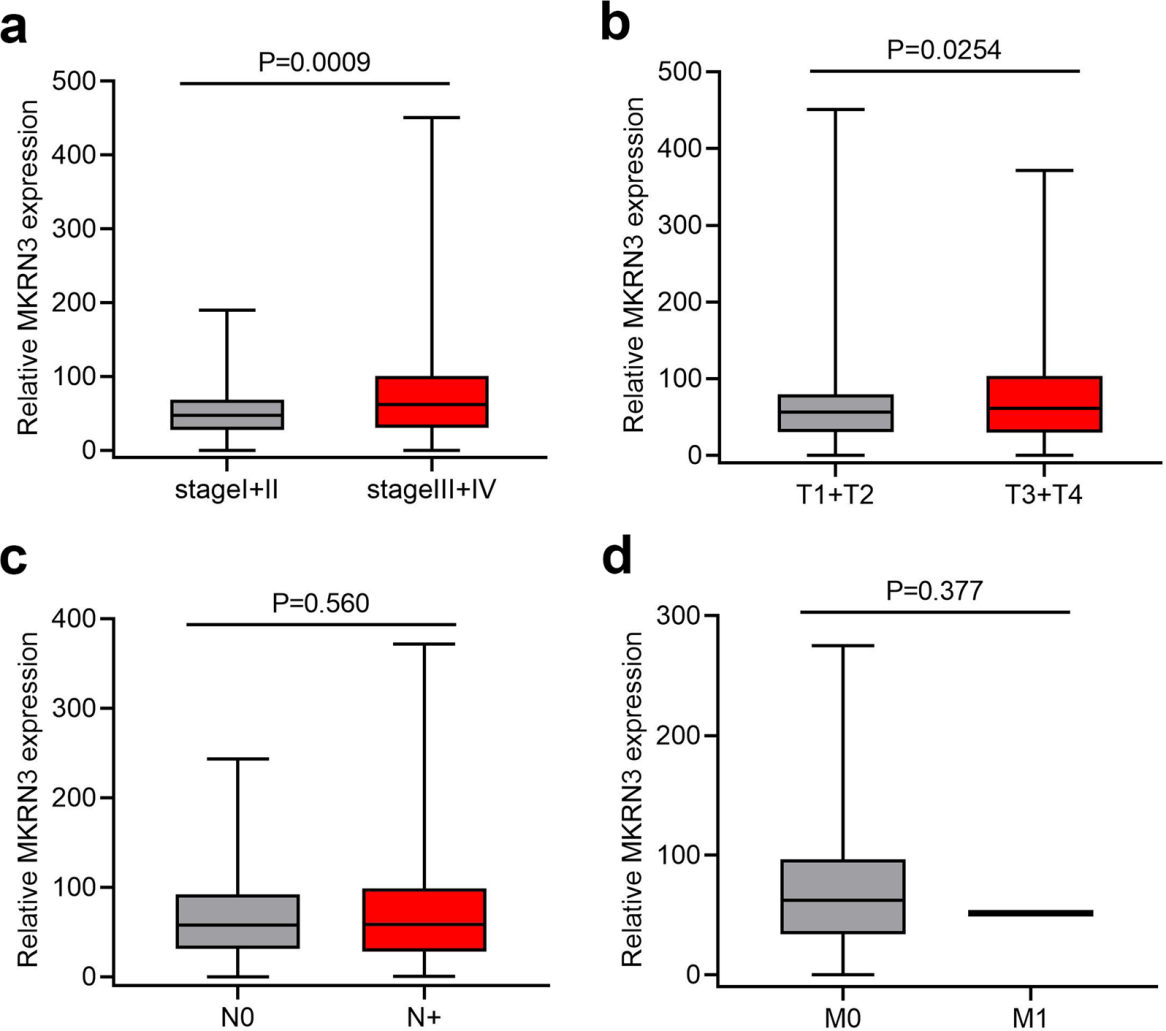
Correlation between MKRN3 expression and clinicopathologic characteristics. **a** Subgroup analysis of clinical stage (stages I + II vs. stages III + IV). **b** T classification (T1 + 2 vs. T3 + T4). **c** N classification (N0 vs. N +). **d** M classification (M0 vs. M1). P-values were calculated using the Mann–Whitney *U* test

**Fig. 5:**
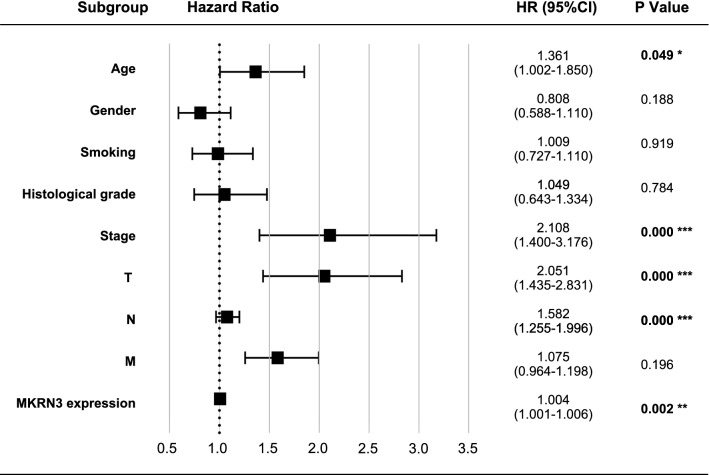
Forest map analysis of MKRN3 expression and clinicopathologic characteristics in SCCHN. P-values were calculated using the Mann–Whitney *U* test. *P < 0.05; **P < 0.01; ***P < 0.001

The survival analysis indicated that patients with high MKRN3 expression levels had a worse prognosis than those with low MKRN3 expression levels in terms of overall survival (520 cases; Fig. 3a; P < 0.01) and disease-free survival (389 cases; Fig. 3b; P < 0.05). Furthermore, the subgroup analysis revealed that high MKRN3 expression was an unfavorable factor for the prognosis of SCCHN patients at stages III + IV (Fig. 4a; P < 0.001) and T3 + 4 (Fig. 4b; P < 0.05), but not in the N (Fig. 4c, P  > 0.05) or M classification (Fig. 4d, P > 0.05). Univariate Cox regression analyses revealed that age, sex, clinical stage, lymph node metastasis, T classification, and status of MKRN3 expression were significantly associated with overall survival (Table 3; all P < 0.05). Additionally, clinical stage, metastasis, T classification, and status of MKRN3 expression were significantly associated with disease-free survival (Table 4; all P < 0.05). Nevertheless, the multivariate Cox regression analyses showed that metastasis, T classification, and MKRN3 expression level were determined to be independent factors with prognostic value for the overall survival (Table 3) and disease-free survival (Table 4) of SCCHN patients. Additionally, the forest plot analysis of overall survival showed statistically significant associations between age, clinical stage, lymph node metastasis, T classification metastasis, and MKRN3 expression and the outcome of SCCHN patients (Fig. 5; all P < 0.05). Collectively, these findings indicate that MKRN3 represents a valuable biomarker in the surveillance and prognosis in SCCHN patients.

**Table 3:** Cox model analysis of overall survival

Parameters	Relative risk (95% CI)	*P*-value
Univariate		
Age	1.318(1.005–1.729)	***0.046***
Gender	0.740(0.557–0.983)	***0.042***
Smoking	1.009(0.767–1.327)	0.949
Alcohol	0.877(0.644–1.194)	0.405
Histological grade	0.828(0.643–1.067)	0.144
Stage	1.767(1.212–2.577)	***0.003***
T classification	1.386(1.114–1.723)	***0.003***
Lymph node metastasis	1.346(1.117–1.622)	***0.002***
Distant metastasis	1.050(0.818–1.348)	0.701
MKRN3 expression	1.004(1.002–1.006)	***0.000***
Multivariate		
Lymph node metastasis	1.721(1.358–2.158)	***0.000***
T classification	1.916(1.389–2.643)	***0.000***
MKRN3 expression	1.004(1.002–1.006)	***0.001***

The bolditalic values represent *P* < 0.05

All the clinicopathological variables listed in the table were included in the univariate and multivariate analyses

95% CI, 95% confidence interval

**Table 4:** Cox model analysis of disease-free survival

Parameters	Relative risk (95% CI)	*P*-value
Univariate		
Age	1.246(0.919–1.691)	0.157
Gender	0.818(0.519–1.137)	0.232
Smoking	0.867(0.643–1.168)	0.348
Alcohol	0.970(0.703–1.337)	0.851
Histological grade	1.036(0.772–1.390)	0.815
Stage	0.541(0.354–0.827)	***0.005***
T classification	2.038(1.439–2.888)	***0.000***
Lymph node metastasis	1.759(1.298–2.483)	***0.000***
Distant metastasis	1.136(1.019–1.266)	***0.022***
MKRN3 expression	1.003(1.001–1.005)	***0.013***
Multivariate		
Lymph node metastasis	1.699(1.215–2.375)	***0.002***
T classification	1.900(1.328–2.718)	***0.000***
Distant metastasis	1.135(1.015–1.269)	***0.027***
MKRN3 expression	1.003(1.001–1.005)	***0.006***

The bolditalic values represent *P* < 0.05

All the clinicopathological variables listed in the table were included in the univariate and multivariate analyses

95% CI, 95% confidence interval

### Functional analysis of MKRN3

**Fig. 6:**
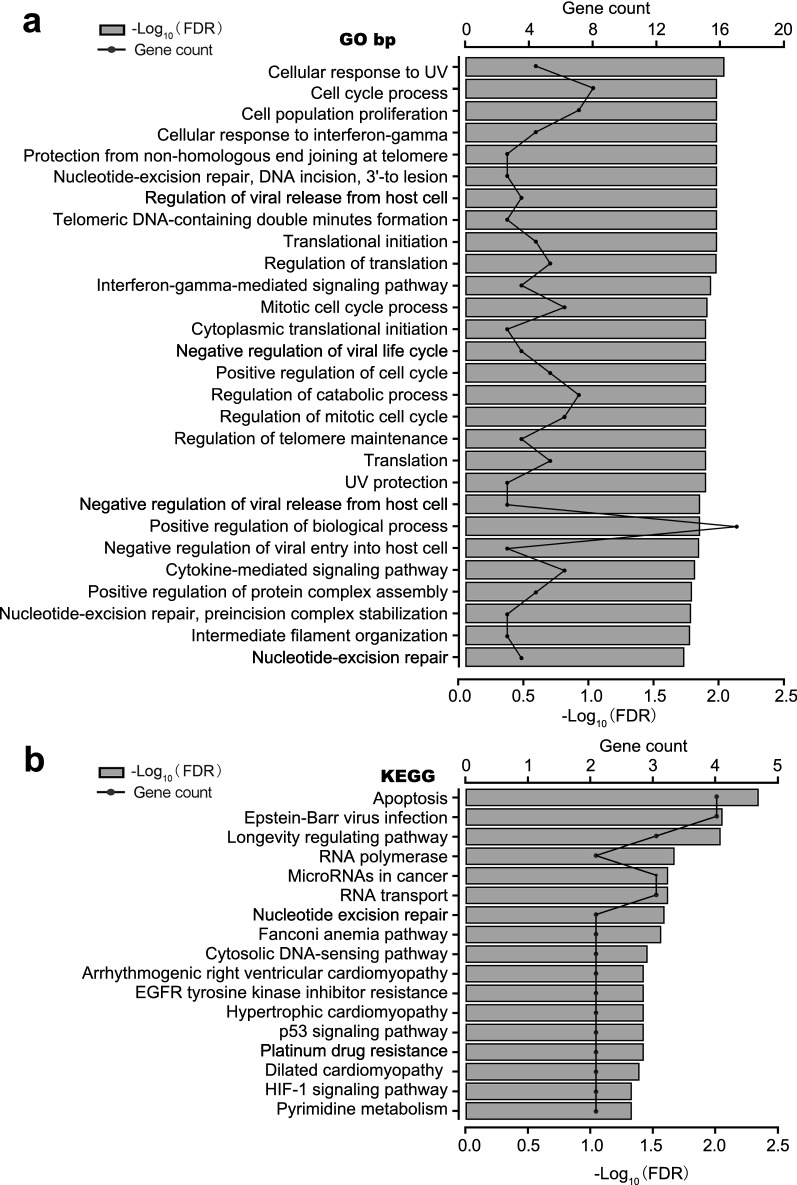
Functional enrichment analysis of MKRN3 in SCCHN. **a** Enriched KEGG biological pathways. **b** Enriched GO terms in the “biological process” category with an FDR < 0.02. The columns indicate different significance levels, and the curve indicates the number of genes. FDR, false discovery rate; GO, gene ontology; KEGG, Kyoto Encyclopedia of Genes and Genomes

**Fig. 7:**
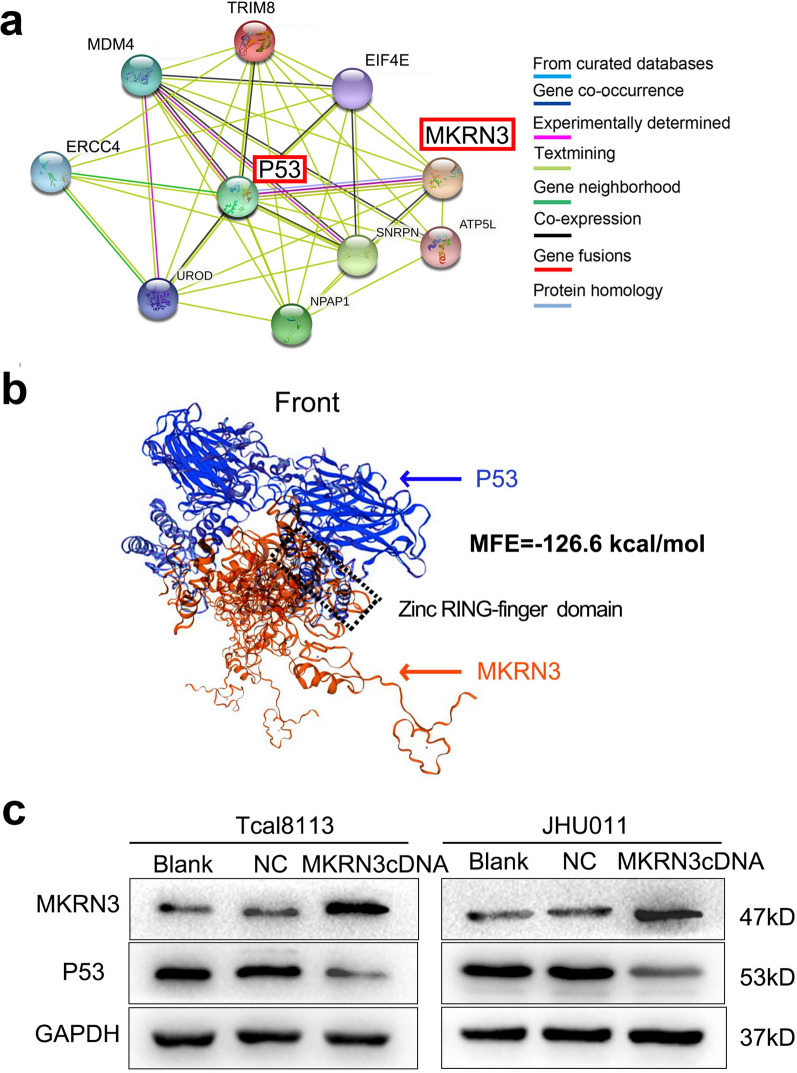
P53 might be a target gene of MKRN3. **a** STRING analysis in the protein–protein interaction of MKRN3. Only the proteins with more than one interaction are displayed. **b** The homologous modeling and molecular docking with MKRN3 and P53. Red and Blue cartoon represent MKRN3 and P53, respectively. The rectangle highlights the interacted domain. **c** Relative expression of P53 protein in Tcal8113 and JHU011 cells that transfected with MKRN3 cDNA and normalized for GAPDH

The functional enrichment clustering of MKRN3 showed that a total of 63 categories from GO biological process, such as DNA synthesis and repair, cellular response to tumor microenvironment, regulation of cellular cycle, and translation were identified as important for cancer development (Fig. 6a; Additional file 3: Table S3). KEGG analysis based on these nodes revealed that 17 pathways in cancer, RNA synthesis and metabolism, and cell growth, death, and motility that were significantly enhanced during SCCHN progression were regulated by MKRN3 (Fig. 6b; Additional file 4: Table S4). These factors were closely related to the occurrence and development of cancer, suggesting that MKRN3 expression exhibits a strong relationship with the progress of SCCHN. Further PPI analysis of MKRN3 illustrated that there were 31 nodes based on a combined score ≥ 0.7 in the STRING analysis, and that P53 might be a direct target gene of MKRN3 (Fig. 7a). Therefore, we used homologous modeling and molecular docking for MKRN3 and P53. As shown in Fig. 7b, the ring finger domain of MKRN3 was supposed to form like a “Goldfish”-like shape and tightly bind with the P53, and the Minimum Free Energy value were − 126.6 kcal/mol. Subsequently, western blot analysis showed that MKRN3 substantially repressed the expression of P53 protein (Fig. 7c). Therefore, our data suggest that P53 might be involved in the MKRN3-mediated SCCHN tumorigenesis.

## Discussion

In this study, we found that elevated MKRN3 expression was correlated with the aggressive tumor characteristics in SCCHN patients, making MKRN3 an independent prognostic predictor for the survival of SCCHN patients. Further functional analysis of MKRN3 provided insight into the biological pathways and mechanisms involved in SCCHN pathogenesis that are regulated by MKRN3. Our data provide evidence that MKRN3 plays an important role in tumor progression and may serve as a critical promoter of SCCHN.

Previous studies investigating MKRN3 has focused on its role as a ubiquitin E3 ligase during puberty initiation [5]. In 2013, mutations with loss of function in the MKRN3 gene were identified from human families with central precocious puberty using whole-exome sequencing [19]. MKRN3 is a maternally imprinted gene located in the Prader–Willi syndrome critical region (chromosome15q11eq13), and only subjects who inherit the mutation from their father develop central precocious puberty [20]. Although MKRN3 is postulated to be an inhibitor of gonadotropin-releasing hormone secretion [21]; the molecular mechanism through which MKRN3 influences the gonadotropin-releasing hormone network remains unclear. In recent years, the correlation between the change in hormonal regulation and cancer has been investigated. Studies have shown that early age at menarche may act as a cancer promoter in breast carcinomas [22], whereas in other types of cancers, the change in hormonal status can inhibit cancer cells invasion [23]. To our knowledge, there was only one report that has evaluated the prognostic value of sex hormone receptor expression in 50 patients with laryngeal squamous cell carcinoma, whose physiological changes occurred due to rapid development during puberty [24]. However, there are few studies on the correlation between MKRN3 expression and tumorigenesis, especially in SCCHN. Thus far, MKRN3 has only been found as an oncogene associated with gastric cancer [25] and imprinted genes in the process of human osteosarcoma [9]. Nevertheless, no research has provided more pervasive evidence to strengthen the links between MKRN3 and tumorigenesis.

With the rapid development of whole-genome sequencing and tumor databases, large-scale global gene expression profiling and database mining becomes more convenient for identifying a potential correlation between gene expression profiles and overall survival in a variety of malignancies [26], including SCCHN [27–30]. The clinical significance of MKRN3, especially its prognostic value in SCCHN, was a key highlight of our current investigation. Herein, we provide evidence that MKRN3 plays a key role in SCCHN progression. Our findings demonstrated that the MKRN3 expression level was markedly increased in SCCHN samples compared to the corresponding adjacent noncancerous tissues. Most importantly, Kaplan–Meyer survival and Cox regression analyses based on the expression level of MKRN3 strengthened the notion that MKRN3 represents a valuable prognosis biomarker with predictive potential in patients with SCCHN. Further studies are required to test whether MKRN3 expression in other solid carcinomas with large patient numbers to broaden its clinical significance.

Consistently, through GO and KEGG functional enrichment analyses, we found that a comprehensive molecular mechanism of MKRN3 action in SCCHN included interferon gamma–mediated signaling pathway, hypoxia-inducible factor 1 signaling pathway, DNA/RNA synthesis and metabolism, and cell cycle regulation. These terms were closely related to the occurrence and development of cancer [31–35]. and further mechanism investigation revealed that P53, a tumor suppressor gene [36], might be a direct target gene of MKRN3. thus, suggesting that MKRN3 is an oncogene and could represent a novel targeted therapeutic strategy for treating SCCHN.

In conclusion, the present study revealed that MKRN3 was upregulated in SCCHN tissues, and its expression may represent a potential marker for prognostic evaluation of SCCHN. However, our study has limitations because the results obtained from bioinformatics analysis are insufficient and need to be confirmed via functional experimental and mechanistic exploration. Furthermore, only one sample out of 522 SCCHN exhibited distant metastases. Therefore, further investigation is required to determine whether MKRN3 may represent an intriguing and novel therapeutic target in SCCHN.

## Conclusion

In this study, we comprehensively analyzed the expression and prognostic values of MKRN3 in SCCHN. We found that MKRN3 possesses a diagnostic value in SCCHN progress. Molecular mechanisms provided important clues for developing novel therapeutic targets in SCCHN and P53 may represent a potential target gene. Overall, MKRN3 might have applications as a prognosis biomarker with predictive potential and a novel therapeutic target in SCCHN patients. Nevertheless, these findings need to be confirmed in the future.

## Supplementary Information

**Additional file 1: Table S1.** The sequence of primers.

**Additional file 2: Table S2.** The antibody information.

**Additional file 3: Table S3.** The GO biological process.

**Additional file 4: Table S4.** The KEGG pathways.

**Additional file 5: Table S5.** The clinical characteristics of 25 SCCHN patients.

**Additional file 6: Table S6.** The data of MKRN3 expression and overall survival.

## Data Availability

The datasets used and/or analyzed during the current study are available from the corresponding author on reasonable request.

## Abbreviations

SCCHN: Squamous cell carcinoma of the head and neck
MKRN3: Makorin ring finger protein 3
TCGA: The Cancer Genome Atlas
IHC: Immunohistochemistry
DMEM: Dulbecco’s modified Eagle medium
GAPDH: Glyceraldehyde-3-phosphate dehydrogenase
SDS-PAGE: Sodium dodecyl sulfatepolyacrylamide gel electrophoresis
PPI: Protein–protein interaction analysis
GO: Gene ontology
KEGG: Kyoto Encyclopedia of Genes and Genomes
