# Correction to: Elevated expression of MKRN3 in squamous cell carcinoma of the head and neck and its clinical significance

**DOI:** 10.1186/s12935-021-02376-y

**Published:** 2021-12-12

**Authors:** Shuiting Zhang, Chao Liu, Guo Li, Yong Liu, Xingwei Wang, Yuanzheng Qiu

**Affiliations:** 1 Department of Otolaryngology Head and Neck Surgery, Xiangya Hospital, Central South University, Changsha, Hunan People’s Republic of China; 2 Department of Anesthesiology, The Second Xiangya Hospital, Central South University, Changsha, Hunan People’s Republic of China; 3 Otolaryngology Major Disease Research Key Laboratory of Hunan Province, Changsha, Hunan People’s Republic of China; 4 Clinical Research Center for Pharyngolaryngeal Diseases, Voice Disorders in Hunan Province, Changsha, Hunan People’s Republic of China

## Correction to: Cancer Cell Int (2021) 21:557 https://doi.org/10.1186/s12935-021-02271-6

In this article [1], the wrong figure appeared as Fig. 7a; the Fig. 7 should have appeared as shown below and the sentence needs to be revised in the “Functional analysis of the Result section” should be:

The sentence currently reads:

Further PPI analysis of MKRN3 illustrated that there were 31 nodes based on a combined score ≥ 0.7 in the STRING analysis, and that P53 might be a direct target gene of MKRN3 (Fig. 7a).

The sentence should read:

Further PPI analysis of MKRN3 illustrated that there were 31 nodes based on a combined score ≥ 0.15 in the STRING analysis, and that P53 might be a direct target gene of MKRN3 (Fig. 7a).

**Fig. 7:**
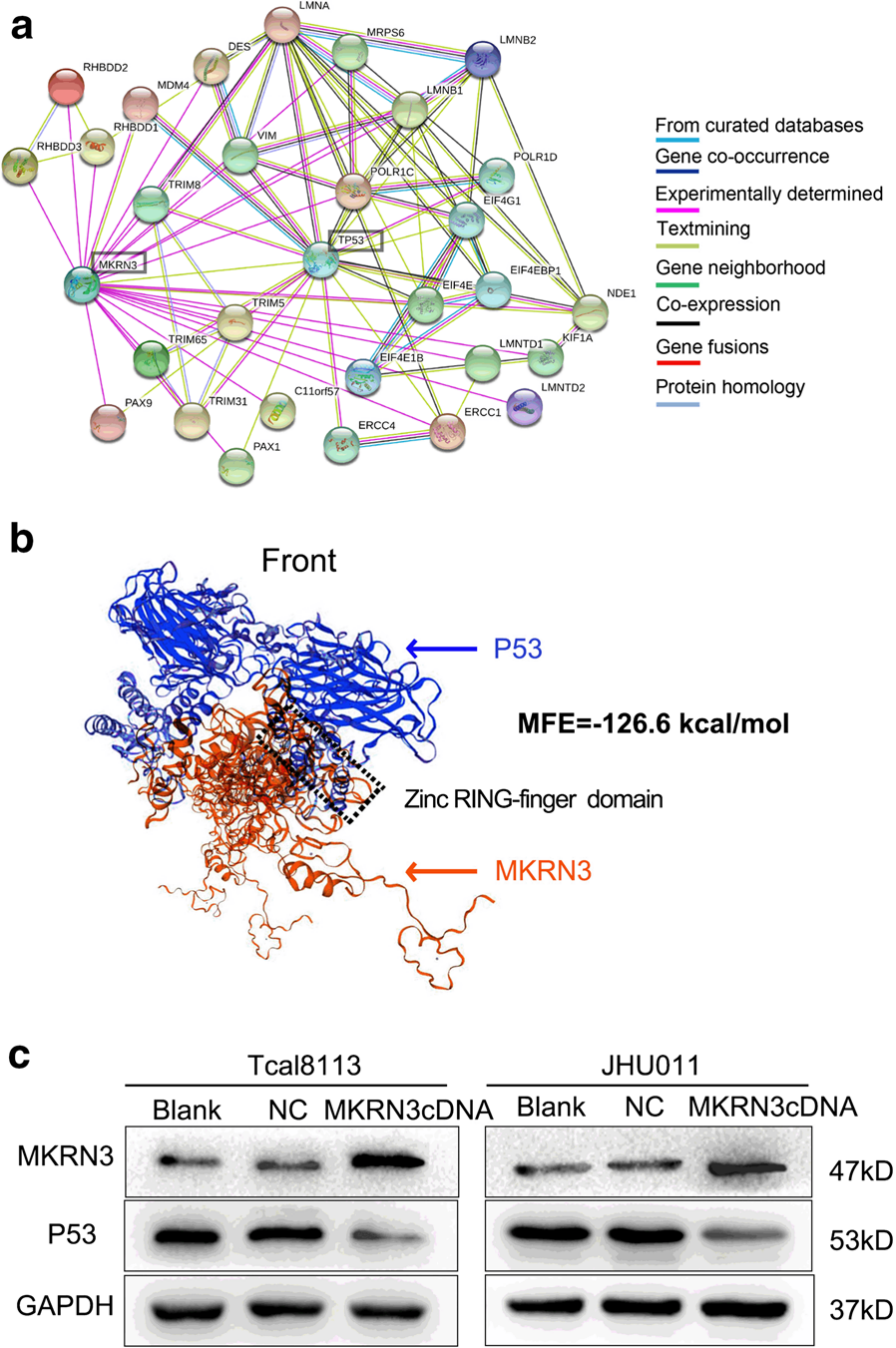
P53 might be a target gene of MKRN3. **a** STRING analysis in the protein–protein interaction of MKRN3. Only the proteins with more than one interaction are displayed. **b **The homologous modeling and molecular docking with MKRN3 and P53. Red and blue cartoon represent MKRN3 and P53, respectively. The rectangle highlights the interacted domain. **c** Relative expression of P53 protein in Tcal1183 and JHU011 cells that transfected with MKRN3 cDNA and normalized for GAPDH
